# Mortality From Ischemic Heart Disease: Analysis of Data From the World Health Organization and Coronary Artery Disease Risk Factors From NCD Risk Factor Collaboration

**DOI:** 10.1161/CIRCOUTCOMES.118.005375

**Published:** 2019-06-04

**Authors:** Alexandra N. Nowbar, Mauro Gitto, James P. Howard, Darrel P. Francis, Rasha Al-Lamee

**Affiliations:** 1 International Centre for Circulatory Health, National Heart and Lung Institute, Imperial College London, Hammersmith Hospital, London, United Kingdom (A.N.N., M.G., J.P.H., D.P.F., R.A.-L.).; 2 Interventional Cardiology Division, Cardio-Thoracic-Vascular Department, San Raffaele Scientific Institute, Milan, Italy (M.G.).

**Keywords:** coronary artery disease, epidemiology, heart diseases, mortality, noncommunicable diseases, risk factors, statistics

## Abstract

**Background:**

Ischemic heart disease (IHD) has been considered the top cause of mortality globally. However, countries differ in their rates and there have been changes over time.

**Methods and Results:**

We analyzed mortality data submitted to the World Health Organization from 2005 to 2015 by individual countries. We explored patterns in relationships with age, sex, and income and calculated age-standardized mortality rates for each country in addition to crude death rates. In 5 illustrative countries which provided detailed data, we analyzed trends of mortality from IHD and 3 noncommunicable diseases (lung cancer, stroke, and chronic lower respiratory tract diseases) and examined the simultaneous trends in important cardiovascular risk factors. Russia, United States, and Ukraine had the largest absolute numbers of deaths among the countries that provided data. Among 5 illustrative countries (United Kingdom, United States, Brazil, Kazakhstan, and Ukraine), IHD was the top cause of death, but mortality from IHD has progressively decreased from 2005 to 2015. Age-standardized IHD mortality rates per 100 000 people per year were much higher in Ukraine (324) and Kazakhstan (97) than in United States (60), Brazil (54), and the United Kingdom (46), with much less difference in other causes of death. All 5 countries showed a progressive decline in IHD mortality, with a decline in smoking and hypertension and in all cases a rise in obesity and type II diabetes mellitus.

**Conclusions:**

IHD remains the single largest cause of death in countries of all income groups. Rates are different between countries and are falling in most countries, indicating great potential for further gains. On the horizon, future improvements may become curtailed by increasing hypertension in some developing countries and more importantly global growth in obesity.

**WHAT IS KNOWN**

- Ischemic heart disease (IHD) is the primary cause of death globally with a recognized set of risk factors.

**WHAT THE STUDY ADDS**

- IHD remains the leading cause of death in countries of all income groups.
- Ukraine and Kazakhstan have particularly high age-standardized mortality rates from IHD, which corresponds to a markedly high prevalence of smoking and hypertension in these countries.
- Although IHD rates are decreasing globally, risk factor prevalence is rising.
- The political and economic transitions that have taken place in Eastern Europe and Central Asia may have contributed to trends in IHD mortality and risk factor control.

Ischemic heart disease (IHD) is the main global cause of death, accounting for >9 million deaths in 2016 according to the World Health Organization (WHO) estimates.^1^ Mortality from IHD in Western countries has dramatically decreased throughout the last decades with greater focus on primary prevention and improved diagnosis and treatment of IHD. However, developing countries pose new challenges for public health. While globalization often improves health care systems, the adoption of Western lifestyles can lead to higher prevalence of cardiovascular risk factors.

We have previously studied the global epidemiology of IHD from 1995 to 2012.^2,3^ In this article, we provide an update, reporting on the burden of IHD mortality up to 2015. Mortality data will be presented by country, sex, and income.

We provide additional country-based analyses of mortality rates by sex, mortality rate changes over time, and risk factors. We display how trends of mortality from IHD in these countries have evolved over the last decade, comparing them with mortality from the major noncommunicable diseases (NCDs) described by the WHO alongside liver disease, infectious disease, and transport accidents as points of reference. Governments worldwide are trying to address risk factors of NCDs.^4^ Therefore, we also analyze how the prevalence of hypertension, smoking, obesity, and diabetes mellitus has changed during these years.

## Methods

### Data Sources

The data that support the findings of this study are available from the corresponding author on reasonable request. The data are drawn from publically available sources, and there is no personally identifiable information about individuals. Therefore, no ethical approval was required for this study.

We used mortality and population data^2,3^ submitted by individual countries to the WHO, in *International Classification of Diseases*-*Ninth Revision* or *International Classification of Diseases*-*Tenth Revision* formats, combined with the United Nations Population Division data query and Gross National Income (GNI) per capita from the World Bank list of economies.^5,6^ However, while other countries may collect vital registration data if countries did not provide data to the WHO, we were unable to include them in the analysis. GNI was defined as the sum of incomes of residents of an economy in a given period. Using the 2017 thresholds, countries were classified into high-income (GNI US $12 057 or higher), upper-middle income (GNI between US $3896 and US $12 056), lower-middle income (GNI between US $996 and US $3895), and low income (GNI US $995 or lower).

Additionally, the NCD Risk Factor Collaboration data set was used to assess the prevalence of hypertension, diabetes mellitus, and the mean adult population body mass index (BMI) in selected countries.^7^ NCD Risk Factor Collaboration is a network of health scientists around the world. Their data set is based on population-based surveys with ≈129 million participants worldwide whose risk factor levels have been measured.

Smoking prevalence data were collected from the WHO Global Health Observatory data repository.^8^ Rates of statin prescription among the adult population in the United Kingdom were taken from the Health Improvement Network primary care data.^9^

Data about prevalence of hypertension, obesity, and type II diabetes mellitus were available for all the countries that had provided IHD mortality data. Data about prevalence of smoking were available for all the countries that had provided IHD mortality data, except Hong Kong SAR, Turkmenistan, and Nicaragua.

### Age-Standardized Mortality Rates

IHD deaths were standardized to the WHO World Standard Population, to allow comparison between populations with a different age distribution.^10^ All the countries that reported mortality data had age information to standardize the rates. Age-standardized mortality rates were calculated using mortality and population data reported within 5-year group ranges. The small number of deaths in the unspecified age group was excluded from this analysis.

### Age-Standardized Mortality Rates Over Time—the 16-Country Analysis

Sixteen countries that had provided the most complete longitudinal data were selected to display trends in age-standardized mortality rates from 2005 to 2015.

### Five-Country Analysis About Sex Differences in Mortality From IHD, NCDs and Risk Factors

Five countries were chosen across a range of geographies and income categories. The United States and United Kingdom were selected as 2 highly populated high-income countries with different healthcare systems and public health strategies. Ukraine, Kazakhstan, and Brazil were then included from low- to high-income categories across a range of continents and healthcare systems.

The prevalence of hypertension, type II diabetes mellitus, and smoking, as well as the mean BMI, were assessed, using the same methods for standardization described above. Hypertension was defined as a systolic blood pressure higher than 140 mm Hg and a diastolic blood pressure lower than 90 mm Hg. Type II diabetes mellitus was defined as fasting plasma glucose of 7.0 mmol/L or higher or history of diabetes mellitus diagnosis or use of insulin or oral hypoglycemic drugs.^7^ For smoking, current tobacco smoking prevalence among people aged 15 years or more was considered. There were no suitable longitudinal data on prevalence of hypercholesterolemia. However, the United Kingdom does report rates of statin prescription.^9^

## Results

### Absolute Numbers of IHD Deaths

The Table shows the total number of deaths caused by IHD worldwide in 2015. For the countries with no mortality data from 2015, we used the data from the most recent available previous year, as long as it was no older than 2005. Among the 98 countries with suitable data, Russia, United States, Ukraine, Germany, and Brazil had the highest total number of deaths. Interestingly, Ukraine had almost the same absolute number of deaths as United States, although Ukraine’s population is one-sixth of the United States. There were limited data on low-income countries, especially those in Africa. India and China also did not provide mortality data for this time period.

**Table.**
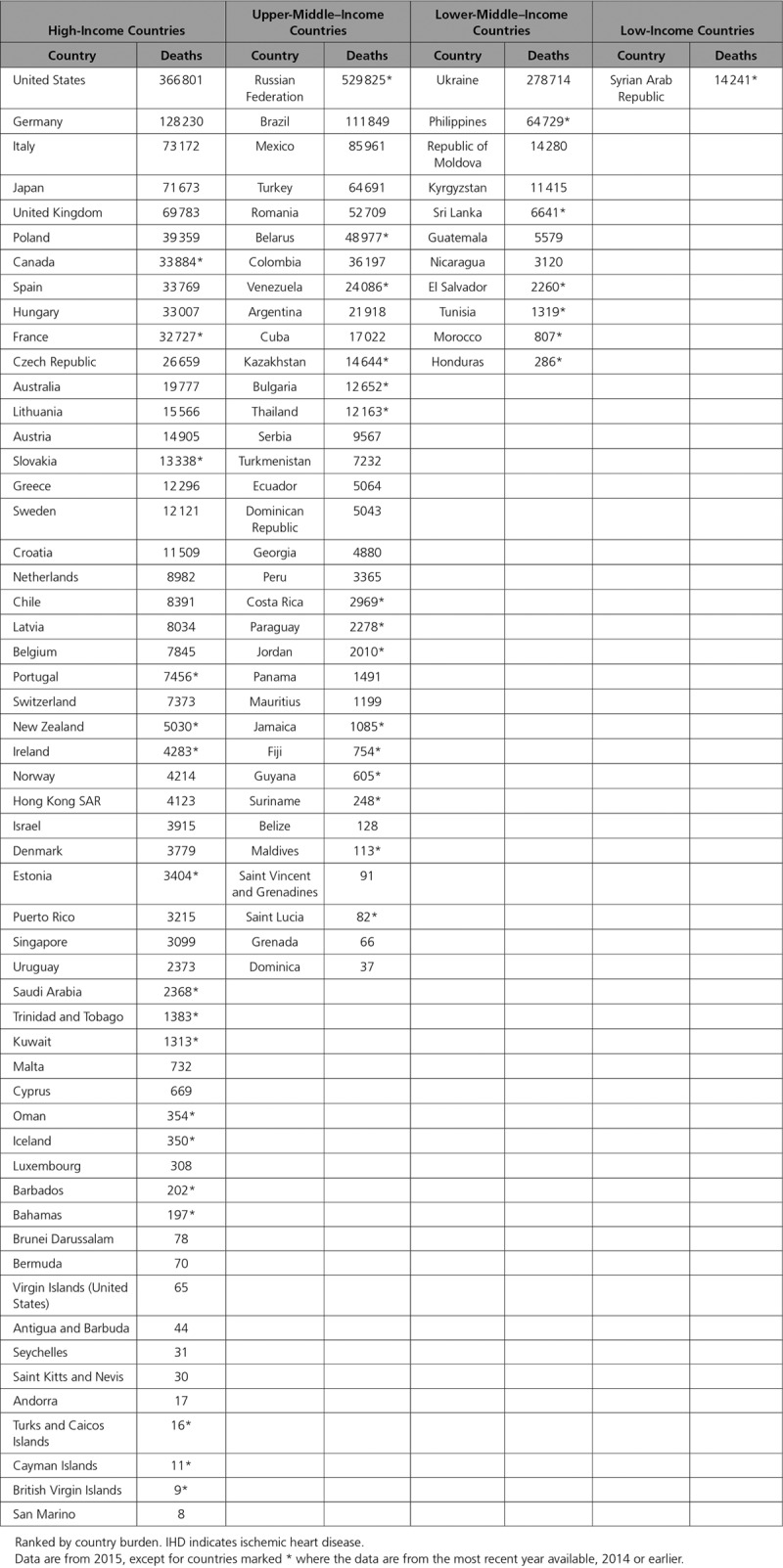
Burden of IHD Deaths From Most Recent Year of Available Data

### IHD Mortality Rates Over Time

**Figure 1.**
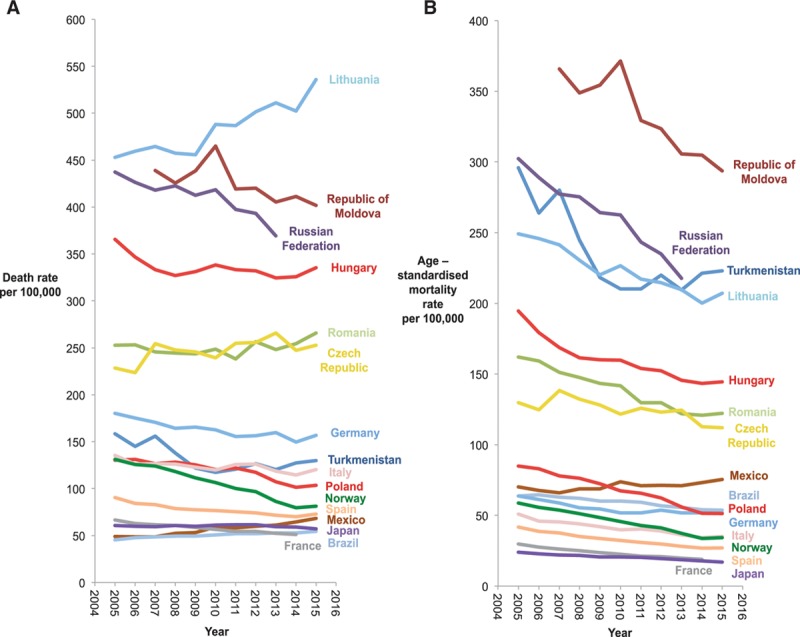
**Changes in (**A**) crude death rates and (**B**) age-standardized mortality rates between 2005 and 2015.** These are the 16 countries who provided longitudinal mortality data.

Figure 1 shows age-standardized and crude mortality rates from 2005 to 2015 in 16 countries for which extensive longitudinal data were available. Lithuania, Republic of Moldova, Russian Federation, Hungary, Romania, and Czech Republic were the countries with the highest mortality from IHD. In each of them, however, both age-standardized and crude mortality rates were declining over time.

### IHD Mortality, Age, and Sex in 5 Selected Countries

**Figure 2.**
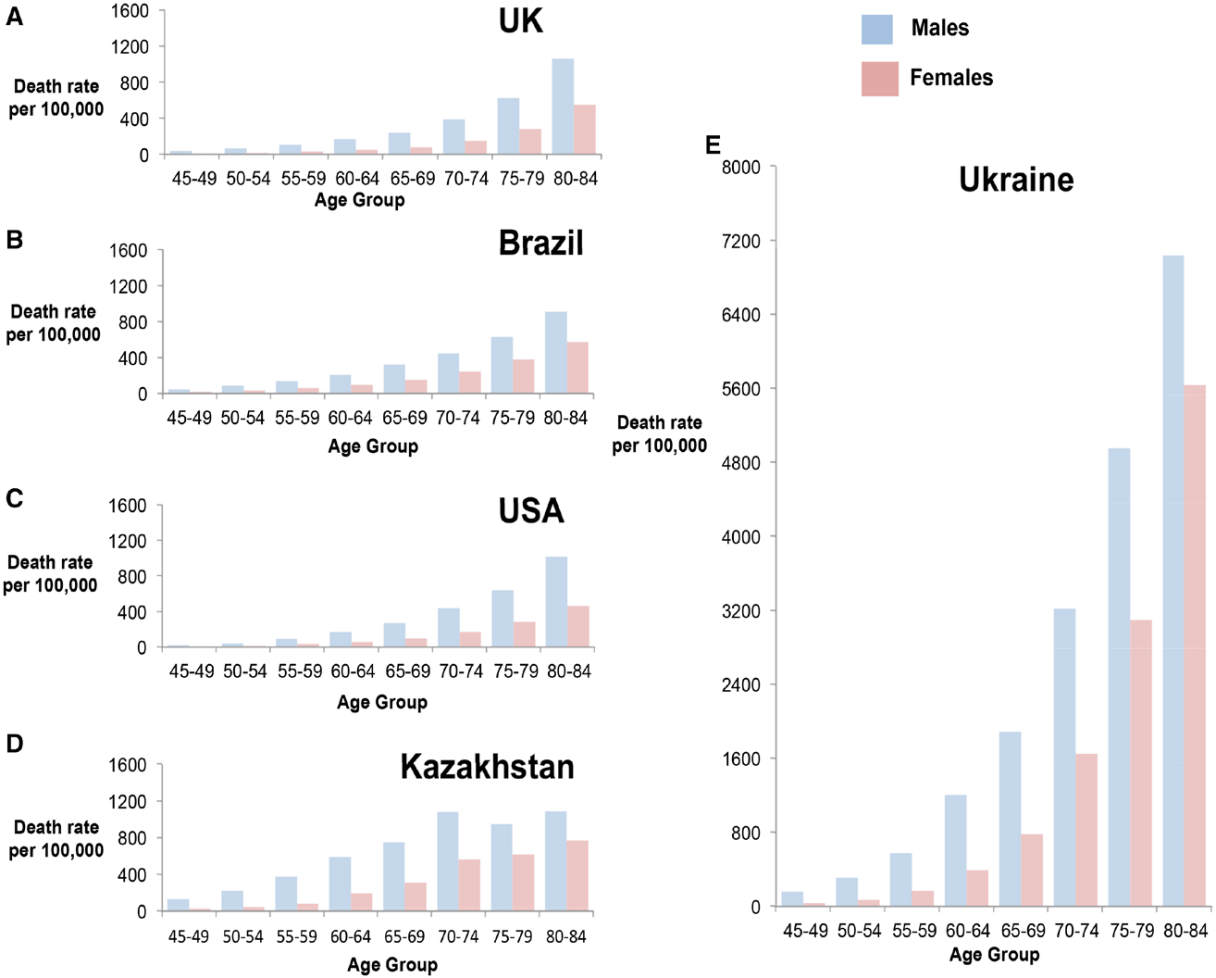
**Variation in age- and sex-specific mortality in (**A**) United Kingdom, (**B**) Brazil, (**C**) United States, (**D**) Kazakhstan, and (**E**) Ukraine.** All data are from 2015 except Kazakhstan which is from 2012.

Figure 2 shows an increase in mortality with age in each of the 5 countries considered (United Kingdom, United States, Ukraine, Kazakhstan, and Brazil). The United Kingdom and United States are high-income countries, Brazil and Kazakhstan are upper-middle income countries, and Ukraine is a lower-middle income country, according to 2017 World Bank classification. They also illustrate a wide geographic distribution and diverse historical backgrounds.

Age-standardized mortality rates increased with age and were generally higher in men than women.

### IHD Mortality and Other NCDs in 5 Selected Countries

**Figure 3.**
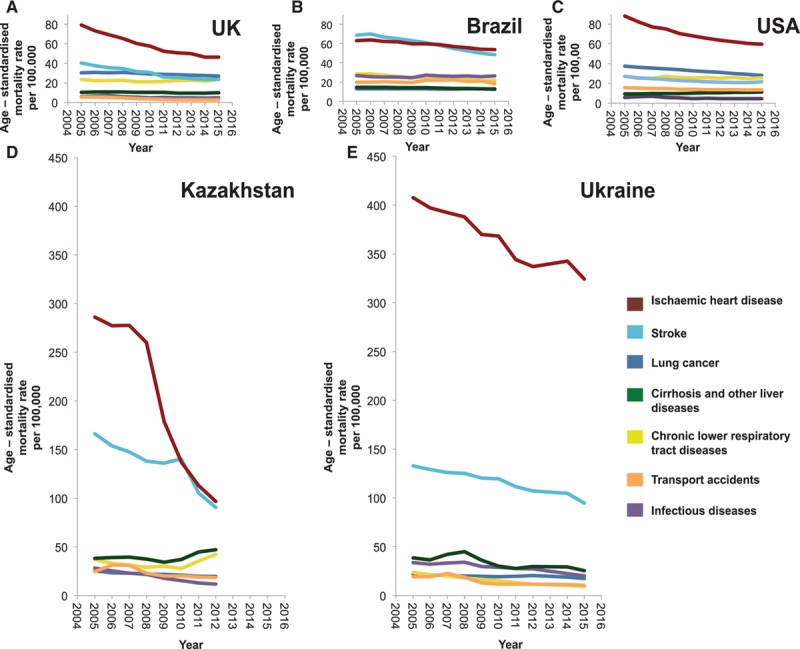
**Mortality trends from major causes of death from 2005 to 2015 in (**A**) United Kingdom, (**B**) Brazil, (**C**) United States, (**D**) Kazakhstan, and (**E**) Ukraine.** Age-standardized mortality rates per 100 000 people from ischemic heart disease (red line), stroke (light blue line), cirrhosis and other liver diseases (green line), chronic lower respiratory tract diseases (yellow line), lung cancer (blue line), transport accidents (orange line), and infectious diseases (purple line).

Figure 3 shows the mortality trends from IHD, the major chronic NCDs (lung cancer, stroke, and chronic lower respiratory tract diseases), cirrhosis and other liver diseases, infectious and parasitic diseases and transport accidents from 2005 to 2015. The temporal trends and relative ranks of these causes of death were widely variable from one country to another.

In the United Kingdom and United States, similar trends were observed. IHD represents the top cause of death, with an age-standardized mortality rate 2- to 3-fold higher the mortality rate for stroke (46 versus 23 in the United Kingdom and 59 versus 21 in United States in 2015). Overall, all-cause mortality was decreasing over time.

Brazil had similar trends to the United Kingdom and United States, but mortality from stroke was comparable to IHD. Initially, stroke accounted for the highest age-standardized mortality rate, before a rapid decrease that led IHD to become the leading cause of mortality from 2010 to 2015.

Although the mortality rate is trending down, IHD is by far the leading cause of death in Ukraine with a strikingly high mortality rate compared with other countries. This is represented by relative size of the figure when drawn to scale (Figure 3). The age-standardized mortality rate from IHD was 3-fold higher than that of stroke and ≈20-fold higher than that of lung cancer.

For Kazakhstan mortality data were only provided until 2012. IHD has remained the top cause of death, but age-standardized mortality rates from IHD decreased from 260 per 100 000 people in 2008 to 97 in 2012. Mortality from stroke had a similar trend during this timeframe. However, mortality from both cirrhosis and chronic lower respiratory tract diseases increased.

### Cardiovascular Risk Factor Prevalence in 5 Selected Countries

**Figure 4.**
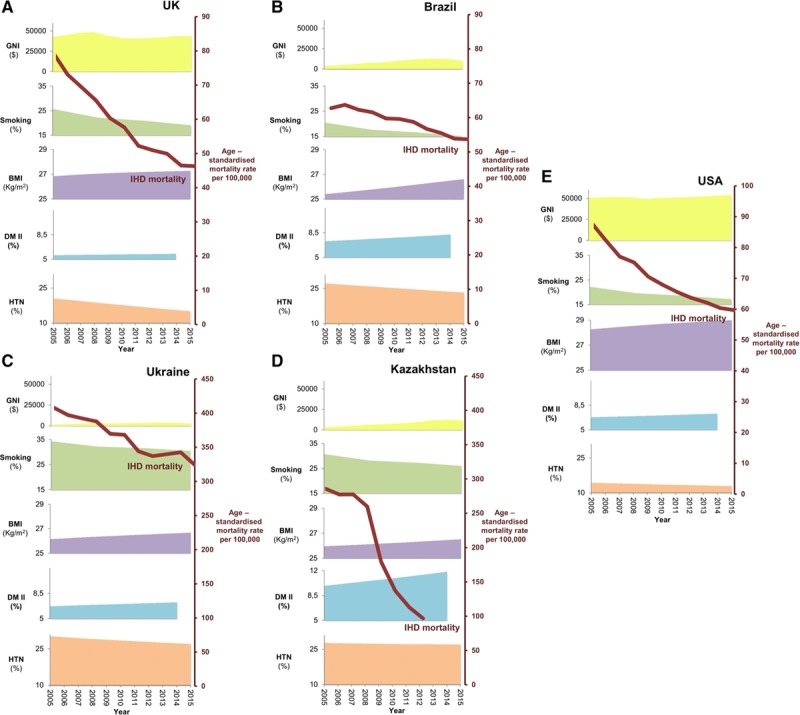
**Mortality trends from ischemic heart disease (IHD) compared with variations in Gross National Income (GNI) and prevalence of cardiovascular risk factors from 2005 to 2015 in (**A**) United Kingdom, (**B**) Brazil, (**C**) United States, (**D**) Kazakhstan, and (**E**) Ukraine.** The red line and right axis represent the IHD mortality trend. Behind this are a family of area charts showing trends of GNI (yellow), age-standardized mean body mass index (BMI; purple), age-standardized prevalence of smoking (green), type II diabetes mellitus (DM II; light blue), and hypertension (HTN; orange).

Figure 4 shows the age-standardized prevalence of 3 cardiovascular risk factors (hypertension, diabetes mellitus, and smoking), the age-standardized mean BMI among adult population and GNI per capita throughout the decade 2005 to 2015. It also displays the IHD age-standardized mortality rate.

Both diabetes mellitus prevalence and mean BMI have been increasing in each of the 5 countries considered above, while the rate of hypertension has decreased in 4 out of 5 countries, remaining almost unchanged in Kazakhstan. Smoking prevalence has also decreased, but it was particularly high in Ukraine and Kazakhstan. GNI increased in Kazakhstan (almost 6-fold higher) and Brazil (almost 3-fold higher) from 2005 to 2015, and despite some minor interruptions of growth, it slightly increased also in the United Kingdom, United States, and Ukraine.

Comparing trends of cardiovascular risk factors and GNI with IHD mortality trends, we observed that the decrease in IHD mortality is not associated with a parallel decrease in the prevalence of cardiovascular risk factors in any of the 5 countries analyzed. Kazakhstan was the country with the most marked reduction in mortality from both IHD and stroke, but also the only one among those selected in which hypertension prevalence has not been decreasing. In Brazil, we observed an increase in mean adult BMI from 25.42 in 2005 to 26.63 in 2015, while IHD age-standardized mortality rate remained relatively stable.

**Figure 5.**
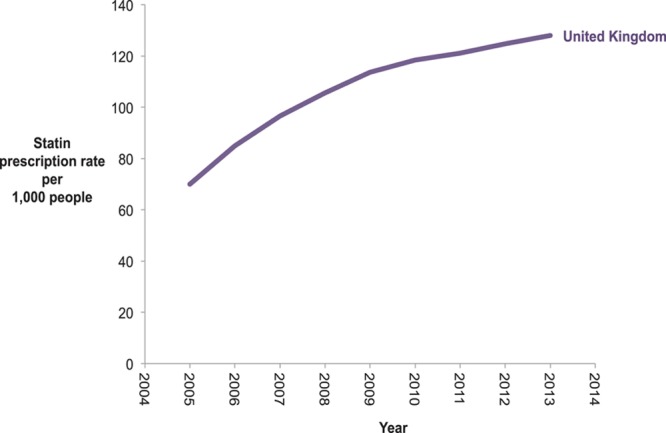
**Statin prescription rates in the United Kingdom from 2005 to 2013.** Rates of prescription in the population over 18 y old.

Figure 5 shows the increase in rates of statin prescription among adult population in the United Kingdom up to 2013. These data are a surrogate because of the lack of raw data available about hypercholesterolemia. It is unknown whether rates of hypercholesterolemia are rising or if detection has increased.

## Discussion

### IHD Mortality Trends

IHD is the top cause of death in countries of all income groups. Mortality trends are slowly but progressively decreasing. This ongoing decrease may be explained by better treatment of cardiovascular risk factors or by an improvement in health care systems.

Healthcare quality improvement often reflects economic growth of a nation.^11^ Especially in the field of heart disease, the availability of advanced diagnostic and therapeutic technologies, such as cardiac catheterization laboratories for coronary angiograms and angioplasties, as well as easy access to drugs are crucial to patients’ management. For each of the 5 countries we looked at, GNI in 2015 was higher than in 2005. Kazakhstan showed the largest rise in income during this period. During the same time frame, risk factors control has not been improving. This suggests economic growth may impact IHD death more than risk factor modification.

Among the 5 countries, age-standardized mortality rates were higher in men compared with women (Figure 2) in all of the age groups. This would support the theory that IHD presents later in women than in men, but we did not formally analyze this relationship.

### Risk Factors

Mortality from NCDs is expected to rise in the coming decades due to worsening of metabolic risk factors. This should result from a worsening of metabolic risk factors, particularly high BMI, diabetes mellitus, hypertension, and high cholesterol. Tobacco consumption is supposed to be decreasing but could easily become the leading risk factor for years of life lost according to the worse health scenarios.^12^ Targeting these risk factors through public health policies may be the best way to interrupt this trend.

Risk factor prevention campaigns have been historically popular in high-income countries. Examples of this include increased taxation on cigarettes, health warnings on tobacco products, banning smoking in public areas, blood pressure testing events in big cities, and mass media campaigns promoting healthy behavior.^13,14^ However, the growing adoption of Western lifestyle may contribute to an increasing prevalence of risk factors in developing countries, where there may be less access to such programs.^15^

The United Kingdom and United States have a lower prevalence of diabetes mellitus and hypertension than Brazil, Kazakhstan, and Ukraine. Mean BMI is highest in United States (out of the 5 countries analyzed), but there is an upward trend of BMI in Brazil. Tobacco control has always been one of the biggest public health challenges and a lot of advocacy interventions to reduce smoking explain the overall decreasing trend.

A cross-sectional study published in 2012 has stated poor awareness of the need for cardiovascular risk factor control in Kazakhstan.^16^ The Global Conference on Primary Health Care, held in Astana in October 2018, reported insufficient primary health care for most developing countries.^15^ Uncontrolled high blood pressure has been described as the leading cause of high IHD burden in former Soviet Union countries. Low adherence to antihypertensive treatments in these countries has been reported. This seems to be because of an insufficient health expenditure that forces patients to out-of-pocket payments to access medications.^17,18^

At the same time, IHD mortality is high even in the United Kingdom and risk factors are likely to play a crucial role in explaining its rates. Interestingly, there is variability among different areas of the nation, with a higher association between risk factors and years of life lost in more deprived socioeconomic areas.^19^ A poor awareness about cardiovascular risk factors in young US adults has also been observed, and those with barriers to health care, such as lack of insurance, were more likely to be unaware.^20^

One way to reduce death from IHD may be to implement public health campaigns focused on primary prevention supported by a primary care infrastructure, extending them both to low- and middle-income countries and to groups with low socioeconomic status in high-income countries. The increasing statin prescription rates in the United Kingdom may indicate an increasing effort of a high-income country to prevent cardiovascular diseases or a rise in prevalence of hypercholesterolemia although this seems less likely.

### Impact of Globalization

We have focused on 5 countries illustrating different steps of globalization. The United Kingdom and United States are a high-income developed countries. In both of them, mortality from IHD, as well as from the other chronic treatable diseases, is progressively decreasing.

In Brazil, an important epidemiological transition has occurred since the 1960s, leading cardiovascular diseases to become the leading cause of mortality. This happened in parallel with urbanization and economic growth.^21^ At present, the profile of mortality from chronic diseases in Brazil is relatively stable, and trends are closer to those observed in United Kingdom rather than in another upper-middle income country, such as Kazakhstan.

In contrast with Brazil, Kazakhstan has more recently undergone globalization. Kazakhstan gained its independence from the Soviet Union in 1991, and since then underwent a rapid growth that led it to become the strongest performing economy in central Asia based on gross domestic product per capita.^22^ This may, in part, be related to being an oil exporter.^22^ Kazakhstan is also the largest country in Central Asia. The rising prevalence of most cardiovascular risk factors is probably the consequence of its political and economic transition, perhaps through unhealthy lifestyle choices like poor diet and lack of exercise. Another explanation might be that improved healthcare led to increased life expectancy allowing time for cardiovascular risk factors to develop. A similar trend has been noted in China.^23^ Kazakhstan was originally a nomad country, and economic development alongside building of modernized towns such as the capital Astana may have promoted the spread of unhealthy lifestyles during the past 2 decades. At the same time, however, increasing wealth is leading to a drastic decrease in age-standardized mortality rates from IHD that have become comparable to those observed in United Kingdom in the last years.

Ukraine is a low-income country, which was part of the Soviet Union until 1991. From the countries who have provided mortality data to WHO, Ukraine has the highest age-standardized mortality rates from IHD. The high IHD mortality rate in Ukraine is in line with other former Soviet Union countries, which have not achieved the improvement in mortality rates seen elsewhere.^17^ While Ukraine is a noticeable outlier, this may be because other low-income countries such as those in Africa have not provided data to the WHO for comparison in this analysis. Poor risk factor control is likely to be contributing to this, as the results concerning smoking and hypertension prevalence have shown. Additionally, several other risk factors, such as alcoholism and psychosocial stress, have been described to play a role in cardiovascular mortality in Eastern Europe.^24^ Thus, both accurate prevention politics and a consistent income growth, not observed in the last decade, would be necessary to address the IHD epidemic in Ukraine.

### Limitations

A major limitation for this analysis is the lack of mortality data for many developing countries. In particular, countries in Africa are under-represented presumably because the systems for data reporting in these countries are underdeveloped. Additionally, data from some large upper-middle and lower-middle income countries, specifically India and China, are unavailable which may limit some of our conclusions pertaining to these particular socioeconomic groups.

The limitation of presenting absolute numbers of deaths is that different countries have different population sizes and a different age-distribution. We, therefore, present crude death rates and age-standardized mortality rates in Figure 1.

For the risk factor analysis, we included only hypertension, smoking, diabetes mellitus, and obesity as we did not have access to data on the prevalence of hypercholesterolemia. These 5 risk factors have been described as the leading contributors to mortality from NCDs in the 2018 GBD Risk Factors Study. However, there are likely to be other risk factors that may variably contribute to mortality from IHD in the different countries, for example, genetic predisposition to IHD.^25^

Additionally, while use of the *InternationalClassification of Diseases* to report causes of death provides standardization, reporting patterns may vary between countries. Different countries may have different methodologies for deciding on cause of death. This may be particularly pertinent in the elderly, where there may be several possible causes of death, therefore, there may be an underestimation or an overestimation of the mortality rates from IHD. This limits the ability to make comparisons between countries at a particular point in time but changes over time within a country should be more reliable as each country is likely to maintain a broadly consistent methodology over time.

Mortality data are drawn from vital statistics, that is, a formal reporting of deaths and causes of death. However, risk factor prevalence was drawn from survey-based data, which is vulnerable to bias through response patterns, and has a larger uncertainty because only a sample is taken, rather than a count across the whole population.

### Conclusions

From WHO mortality data updated to 2015, IHD remains the leading cause of death in countries of all income groups. However, while IHD mortality is falling globally, mortality rates in many countries, particularly those in lower- and middle-income brackets, remain very high. The prevalence of cardiovascular risk factors continues to rise. Globalization seems to have contributed to a higher prevalence of risk factors in developing countries. Improvement in primary prevention strategies and implementation of public health policies are needed to reduce worldwide mortality from this disease.

## Sources of Funding

J.P. Howard is supported by the Wellcome Trust (212183/Z/18/Z). A.N. Nowbar acknowledges support from the National Institute for Health Research Imperial Biomedical Research Centre (P74227).

## Disclosures

Dr Al-Lamee receives speaker’s honoraria from Phillips Volcano. The other authors report no conflicts.
